# A Single Amino Acid Replacement Boosts the Analgesic Activity of α-Conotoxin AuIB through the Inhibition of the GABA_B_R-Coupled N-Type Calcium Channel

**DOI:** 10.3390/md20120750

**Published:** 2022-11-29

**Authors:** Yuanmei Wei, Min Zhang, Shuo Yu, Qiuyuan Huang, Rongfang Chen, Shujing Xu, Yue Huang, Yunzhou Yu, Ming Liao, Qiuyun Dai

**Affiliations:** 1 Beijing Institute of Biotechnology, Beijing 100071, China; 2 Institute of Snake Venom, School of Basic Medical Sciences, Guangxi Medical University, Nanning 530021, China

**Keywords:** α-conotoxins AuIB, structure–activity relationship, GABA_B_R-coupled Ca_V_2.2, analgesic activity

## Abstract

α-conotoxin AuIB is the only one of the 4/6 type α-conotoxins (α-CTxs) that inhibits the γ-aminobutyric acid receptor B (GABA_B_R)-coupled N-type calcium channel (Ca_V_2.2). To improve its inhibitory activity, a series of variants were synthesized and evaluated according to the structure–activity relationships of 4/7 type α-CTxs targeting GABA_B_R-coupled Ca_V_2.2. Surprisingly, only the substitution of Pro7 with Arg results in a 2–3-fold increase in the inhibition of GABA_B_R-coupled Ca_V_2.2 (IC_50_ is 0.74 nM); substitutions of position 9–12 with basic or hydrophobic amino acid and the addition of hydrophobic amino acid Leu or Ile at the second loop to mimic 4/7 type α-CTxs all failed to improve the inhibitory activity of AuIB against GABA_B_R-coupled Ca_V_2.2. Interestingly, the most potent form of AuIB[P7R] has disulfide bridges of “1–4, 2–3” (ribbon), which differs from the “1–3, 2–4” (globular) in the isoforms of wildtype AuIB. In addition, AuIB[P7R](globular) displays potent analgesic activity in the acetic acid writhing model and the partial sciatic nerve injury (PNL) model. Our study demonstrated that 4/6 type α-CTxs, with the disulfide bridge connectivity “1–4, 2–3,” are also potent inhibitors for GABA_B_R-coupled Ca_V_2.2, exhibiting potent analgesic activity.

## 1. Introduction

α-Conotoxins (α-CTxs) are a big superfamily of conotoxins, which are derived from the venom of marine *Conus* snails, mainly inhibiting nicotinic acetylcholine receptors (nAChRs) [1,2]. α-CTxs consist of 12–20 amino acid residues, with 2 or 3 disulfide bonds. According to the residue numbers of the inter cysteine loops (-CC-(loop1)-C-(loop2)-C-), α-CTxs are divided into several subfamilies, such as α3/5, α4/3, α4/4, α4/6, α4/7, and α5/5 [3,4]. Among all α-CTxs, α4/7 CTxs are the most common. Functionally, some α4/7 CTxs have been found to potently inhibit nAChRs subtypes, such as α9α10 [5,6] and α3β2 [7]. In addition, a few α4/7 and 4/3 CTxs, such as Vc1.1 [8,9,10,11,12], PeIA [13,14,15,16], RgIX [17,18,19,20], Lt1.3 [21], and Eu1.6 [22], were also found to inhibit the GABA_B_R-coupled N-type calcium channels (Ca_V_2.2) or Ca_V_2.2 alone, with the latter target recognized as the main source of analgesic activity [23]. In contrast, significantly fewer α4/6 CTxs have been found, and only two 4/6-type α-conotoxin AuIB and ViIA have been found to inhibit α3β4 [10,24,25], α3β2 nAChR [26], and GABA_B_R-coupled Ca_V_2.2 [27] (Table 1). AuIB was originally derived from the venom of *Conus aulicus* and consists of 15 amino acid residues, as well as 2 disulfide bonds [24]. Its analgesic activity has been recognized to originate from the inhibition of GABA_B_R-coupled Ca_V_2.2, since it only displays modest inhibition of the α3β4 nAChR subtype [27,28]. To date, no study of its structure–activity relationship has been reported regarding its inhibition of GABA_B_R-coupled Ca_V_2.2.

To improve the potency of 4/6-type α-conotoxins against GABA_B_R-coupled Ca_V_2.2, in the present study, a series of AuIB variants were designed and synthesized according to the structure–activity of 4/7 type α-CTxs (Table 2) targeting GABA_B_R-coupled Ca_V_2.2 [11,12,14]. The inhibitory activity of variants against GABA_B_R-coupled Cav2.2 or Ca_V_2.2 alone, expressed on HEK293, was evaluated using electrophysiological methods. The analgesic activities of AuIB and the most potent variant were also determined using the acetic acid writhing model, the hot plate model, and the partial sciatic nerve injury (PNL) model. We were surprised to find that only the substitution of Pro7 with Arg was able to increase the inhibitory activity of AuIB against GABA_B_R-coupled Ca_V_2.2 (IC_50_ is 0.74 nM). The AuIB variants with substitutions of amino acid at position 9–12 with basic or hydrophobic amino acid, as well as the addition of hydrophobic amino acid Leu or Ile at the second loop as 4/7 type α-CTxs, did not improve the inhibitory activity. The most potent isoform, AuIB[P7R], displays no significant inhibitory activity against nAChRs, including the α3β4 subtype. In addition, AuIB[P7R] is the ribbon isomer with the disulfide bridges “1–4, 2–3,” but not “1–3, 2–4”(globular), as in wildtype AuIB. Finally, AuIB[P7R](ribbon) displays potent analgesic activity in the acetic acid writhing model and the partial sciatic nerve injury (PNL) model. Our study provides a new potent GABA_B_R-coupled Ca_V_2.2 inhibitor, demonstrating that 4/6 type α-CTx, with the disulfide bridge connectivity “1–4, 2–3,” is also a potent motif for designing potent inhibitors of GABA_B_R-coupled Ca_V_2.2.

## 2. Results

### 2.1. Synthesis and Characterization of AuIB and Its Variants

**Figure 1:**
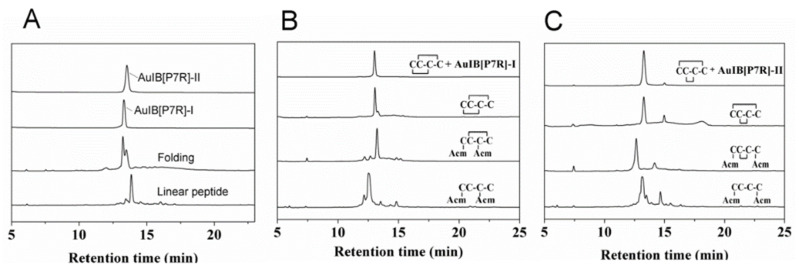
HPLC analyses of folded products of linear and Acm-modified AuIB[P7R]. (**A**) Folded products of linear AuIB[P7R]: traces from bottom to top: linear peptide; one-step folded products; pure AuIB[P7R]-I; pure AuIB[P7R]-II. (**B**,**C**) Determination of the disulfide bond connectivity of AuIB[P7R]-I (**B**) and AuIB[P7R]-II (**C**): traces from bottom to top: linear peptide with Acm modifications at Cys1 and Cys3 or Cys 1 and Cys 4; the first oxidized products; the second oxidized products; co-elution of the two-step folding products plus the purified product AuIB[P7R-I] or AuIB[P7R]-II. Samples were applied to an Agilent Eclipse Plus C18 column (5 μm, 4.6 mm × 250 mm) and eluted with a linear gradient of 0–10% B for 0–1 min; 10–50% B (B is acetonitrile containing 0.1% trifluoroacetic acid (TFA)) for 1–25 min. Absorbance was monitored at 214 nm. The flow rate was 1.0 mL/min.

Most linear 4/6-type AuIB and its variants fold into two main products in 0.1 M NH_4_HCO_3_ buffer (pH 8.0–8.2). The analyses of the folding products of AuIB[P7R] is shown in Figure 1A. All folding products (Table 1) were purified and assessed with analytical reversed-phase HPLC, and the purity of the peptides was greater than 95%. All peptides exhibited the expected molecular weights, as ascertained by mass spectrometry.

The disulfide bond connectivity of AuIB was confirmed, according to the methods of previous studies [21]. The disulfide bond connectivity of the AuIB mutant AuIB[P7R] was determined using the two-step synthesis method. The HPLC results of the one-step and two-step folding of the acetamidomethyl (Acm)-protected linear peptides are shown in Figure 1B,C. The disulfide bond connectivity of the AuIB[P7R]-I peak is “C1-C3, C2-C4” (globular, Figure 1B), and the disulfide bond connectivity of AuIB[P7R]-II is “C1-C4, C2-C3” (ribbon, Figure 1C). The elution time is different from that of wild type AuIB; the isomer AuIB(ribbon) was eluted first, but AuIB(globular) was eluted later [29].

To improve the potency of the AuIB variants, a series of 4/6 type AuIB variants 2–18 (Table 1) were synthesized according to the structure–activity of 4/7 type α-CTxs targeting GABA_B_R-coupled Ca_V_2.2 (Table 2), in which a basic acid at position 4, 7, 9, and 10, or an acidic amino acid at position 4 and 11, or the hydrophobic amino acid benzoyl group introduced at the N-terminus of Vc1.1 could enhance the potency [12,14,27]. In addition, 4/7 type AuIB variants 19–26 were also synthesized by the addition of a hydrophobic acid Leu at position 15.

**Table 1:** Amino acid sequence and inhibitory activity of AuIB and its variants against GABA_B_R-coupled Ca_V_2.2.

NO.	Name	Amino Acid Sequence	Inhibition Efficacy (1 µM)%	IC_50_ (nM) (95% Confidence Interval)
**1**	AuIB(ribbon)	G**CC**SYPP**C**FATNPDC*	9.28 ± 2.65	
**2**	AuIB(globular)	G**CC**SYPP**C**FATNPDC*	27.40 ± 2.05	1.93 (0.42–8.86)
**3**	Benzoyl-AuIB(ribbon)	Benzoyl-G**CC**SYPP**C**FATNPDC*	9.00 ± 1.64	
**4**	Benzoyl-AuIB(globular)	Benzoyl -G**CC**SYPP**C**FATNPDC*	18.93 ± 2.88	
**5**	AuIB[S4Dab]-I	G**CC*Dab-***YPP**C**FATNPDC*	8.11 ± 1.99	
**6**	AuIB[S4Dab]-II	G**CC*Dab-***YPP**C**FATNPDC*	25.84 ± 2.73	
**7**	AuIB[P7R](globular)	G**CC**SYP***R*C**FATNPDC*	17.12 ± 0.70	
**8**	AuIB[P7R](ribbon)	G**CC**SYP***R*C**FATNPDC*	51.58 ± 1.86	0.74 (0.26–2.09)
**9**	Benzoyl-AuIB[P7R](globular)	Benzoyl-G**CC**SYP***R*C**FATNPDC*	18.70 ± 5.25	
**10**	Benzoyl-AuIB[P7R](ribbon)	Benzoyl -G**CC**SYP***R*C**FATNPDC*	23.62 ± 3.26	
11	AuIB[F9Y]-I	G**CC**SYPP**C*Y***ATNPDC*	8.38 ± 1.99	
12	AuIB[F9Y]-II	G**CC**SYPP**C*Y***ATNPDC*	8.28 ± 1.96	
13	AuIB[F9R]	G**CC**SYPP**C*R***ATNPDC*	12.58 ± 1.99	
14	AuIB[A10R]-I	G**CC**SYPP**C**F***R***TNPDC*	8.46 ± 1.12	
15	AuIB[A10R]-II	G**CC**SYPP**C**F***R***TNPDC*	21.50 ± 4.71	
16	AuIB[T11D]	G**CC**SYPP**C**FA***D***NPDC*	27.96 ± 3.01	
17	AuIB[N12H]-I	G**CC**SYPP**C**FAT***H***PDC*	15.29 ± 1.67	
18	AuIB[N12H]-II	G**CC**SYPP**C**FAT***H***PDC*	14.11 ± 2.17	
19	AuIB[+15I]	G**CC**SYPP**C**FATNPD*I*C*	30.38 ± 3.26	52.28 (11.16–245.0)
20	AuIB[+15L]	G**CC**SYPP**C**FATNPD*L*C*	27.85 ± 1.37	25.45 (7.35–88.06)
21	AuIB[A10R,+15L]-I	G**CC**SYPP**C**F***R***TNPD*L*C*	24.17 ± 2.38	
22	AuIB[A10R,+15L]-II	G**CC**SYPP**C**F***R***TNPD*L*C*	18.49 ± 1.01	
23	AuIB[Y5D,+15L]	G**CC**S***D***PP**C**FATNPD*L*C*	17.62 ± 1.98	
24	AuIB[Y5D,F9R,+15L]	G**CC**S***D***PP**C*R***ATNPD*L*C*	14.42 ± 1.27	
25	AuIB[P7R,+15L]-I	G**CC**SYP***R*C**FATNPD*L*C*	14.54 ± 1.10	
26	AuIB[P7R,+15L]-II	G**CC**SYP***R*C**FATNPD*L*C*	19.44 ± 2.39	

* C-terminus is amidated; Cysteine font is bold, mutation site font is bold and italic.

**Table 2:** α-Conotoxins targeting GABA_B_R-coupled Ca_V_2.2. *: C-terminus amidated; Cysteine font is bold.

α-Contoxin	Subfamily	Amino Acid Sequence	IC_50_(nM)	Reference
Vc1.1	4/7	G**CC**SDPR**C**NYDHPEIC*	2.38	[27]
Vc1.1[N9R]	4/7	G**CC**SDPR**C**RYDHPEIC*	4.87	[12]
Benzoyl-Vc1.1[N9R]	4/7	Benzoyl-G**CC**SDPR**C**RYDHPEIC*	0.19	[12]
Vc1.2	4/7	G**CC**SNPA**C**MVNNPQIC*	47.5	[14]
PeIA	4/7	G**CC**SHPA**C**SVNHPELC*	1.1	[14]
Lt1.3-I	4/7	G**CC**SHPA**C**SGNNPYFC*	33.9	[21]
RgIA	4/3	G**CC**SDPR**C**RYR**C**R*	7.3	[17]
AuIB(globular)	4/6	G**CC**SYPP**C**FATNPDC*	1.5(1.93)	[27] (This work)
AuIB[P7R](ribbon)	4/6	G**CC**SYP***R*C**FATNPDC*	0.74	This work

Compared to 4/6 type AuIB variants, 4/7-type AuIB variants mainly formed a single primary product (data not shown), several AuIB variants, such as AuIB[A10R,+15L] and AuIB[P7R,+15L], also form two products [29].

### 2.2. Circular Dichroism of α-Conotoxin AuIB Variants

**Figure 2:**
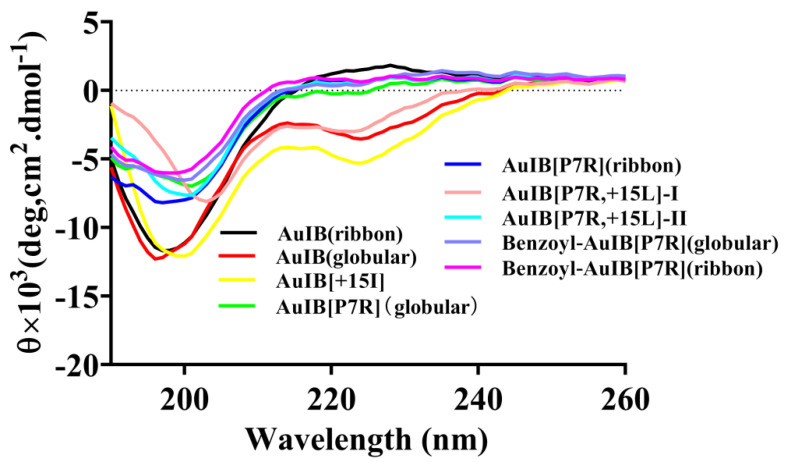
Circular dichroism spectra of AuIB and its variants. The peptide was dissolved in 0.01 M PBS (pH = 7.2). λ = 190~260 nm; d = 1 mm; *n* = 3.

To determine if the difference in the potency of the AuIB variants resulted from their structures, we acquired the typical CD spectra of AuIB and its typical variants, shown in Figure 2. The results show that AuIB(globular) with the disulfide bond connectivity “C1–C3, C2–C4” and the 4/7-type AuIB variants have a low α-helix content, while others, including AuIB[P7R](globular) and AuIB[P7R](ribbon) possess no apparent α-helical structure.

### 2.3. Inhibitory Effects on GABA_B_R-Coupled Ca_v_2.2 Currents Induced by AuIB and Its Variants

**Figure 3:**
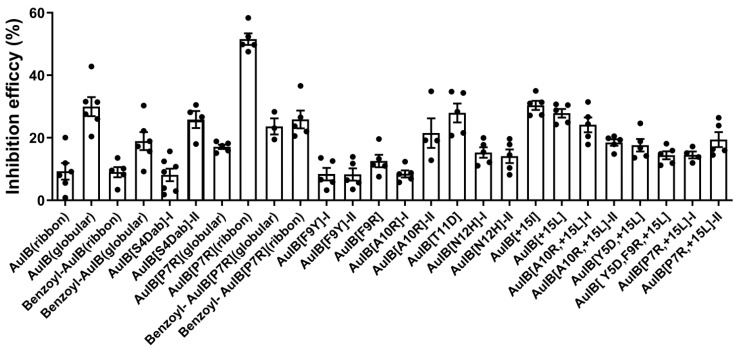
Inhibitory efficacy ((1 − I/I_0_) × 100%) of 1 μM AuIB and its variants on GABA_B_R-coupled Ca_V_2.2 channels. All data are presented as mean ± SEM; *n* ≥ 4.

**Figure 4:**
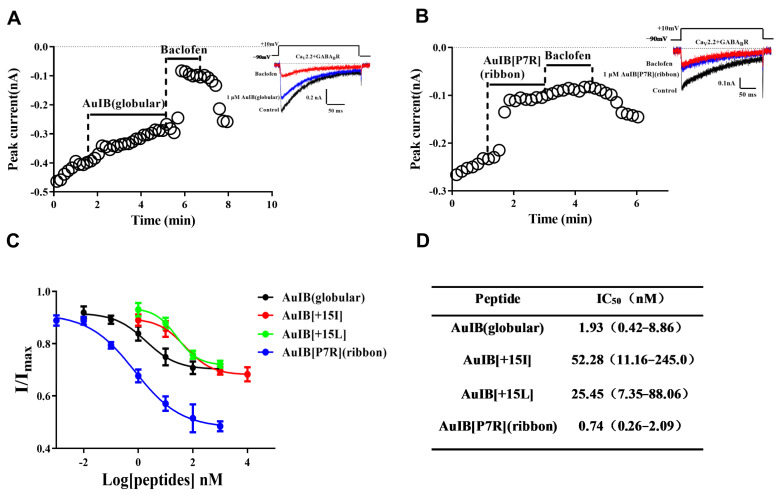
Concentration-response analysis of the activity of AuIB and its typical variants on the GABA_B_R-coupled Ca_V_2.2 channels. (**A**,**B**) Representative traces of whole-cell currents, in the presence of 1 M AuIB (**A**) and AuIB[P7R](ribbon) (**B**). (**C**) Concentration–response curves obtained for inhibition of GABA_B_R-coupled Ca_V_2.2 channels by AuIB and its variants. (**D**) IC_50_ values of AuIB and its variants. Currents were recorded by the whole-cell patch clamp of HEK293 cells (n ≥ 4). All data are presented as mean ± SEM.

The inhibitory activity of 1 μM of AuIB and its variants at the GABA_B_R-coupled Ca_V_2.2 channels was determined in the HEK293T cells (Figure 3). The results show that AuIB[P7R](ribbon) displays the highest potency; the inhibition efficacy is 51.58%, significantly higher than that of the other AuIB variants. AuIB(globular), AuIB[S4Dab], AuIB[T11D], AuIB[+15L], and AuIB[+15I] also exhibit potent inhibitory activity, with inhibition efficacy from 25.84–30.38%, but all others display lower potency (<24.17%). The concentration–response curves of AuIB(globular), AuIB[P7R](ribbon), AuIB[+15L], and AuIB[+15I] are shown in Figure 4C,D. Compared to wild type AuIB(globular), AuIB[P7R](ribbon) has a lower IC_50_ value (0.74 (0.26–2.09) nM), less than half of AuIB(globular) (IC_50_ = 1.93 (0.42–8.86) nM). However, other AuIB variants, AuIB[+15I] and AuIB[+15L], show significantly lower inhibitory activity, with IC_50_ values of 52.28 (11.16–245.0) nM and 25.45 (7.35–88.06) nM, respectively. Importantly, AuIB[P7R](ribbon) could produce a sharp decrease in peak currents, which was demonstrated in the representative traces of GABA_B_R-coupled Ca_v_2.2 channels depolarization-activated Ba^2+^ currents inhibited by 1 μM AuIB(globular) and AuIB[P7R](ribbon) (Figure 4A,B).

In addition, the direct inhibitory effect of 10 μM AuIB[P7R](ribbon) on Ca_V_2.2 was determined to be 14.87 ± 2.42%, much lower than that of GABA_B_R-coupled Ca_v_2.2.

### 2.4. Inhibitory Activity of AuIB[P7R](ribbon) against Isoforms of Neuronal nAChRs

AuIB[P7R](ribbon) and AuIB[P7R](globular) were further tested for their inhibitory activity on the Ach-evoked current amplitude mediated by various rat nAChR subtypes (α2β4, α3β4, α4β4, α7, and α9α10) expressed in *Xenopus* oocytes. As shown in Table 3, 10 µM AuIB[P7R](ribbon) and AuIB[P7R](globular) exhibit low inhibitory activities against nAChRs subtypes, and the inhibition rate of both AuIB variants is below 50%, indicating that AuIB[P7R](ribbon) is a selective GABA_B_R-coupled Ca_V_2.2 inhibitor.

**Table 3:** Inhibitory effects of 10 µM AuIB[P7R](globular) and AuIB[P7R](ribbon) on various neuronal nAChR subtypes.

Subtype	AuIB[P7R](ribbon) (%)	AuIB[P7R](globular) (%)
α2β4	39.43 ± 6.31	34.95 ± 9.69
α3β2	29.25 ± 12.65	5.59 ± 4.48
α3β4	39.27 ± 6.45	6.62 ± 5.75
α4β2	35.92 ± 12.50	8.87 ± 7.19
α4β4	30.44 ± 8.03	5.48 ± 6.28
α7	17.00 ± 0.35	27.83 ± 10.00
α9α10	5.09 ± 7.85	21.32 ± 12.17

All data are presented as mean ± SEM. *n* ≥ 4. IC_50_ of AuIB(globular): >>1 µM (α9α10, α3β2), 0.75 µM (α3β4) [27]; AuIB-I(ribbon): inhibition efficacy ~50% (10 µM) [29,30].

### 2.5. AuIB[P7R](ribbon) Significantly Reduces the Writhing Number in the Acetic Acid Writhing Model

**Figure 5:**
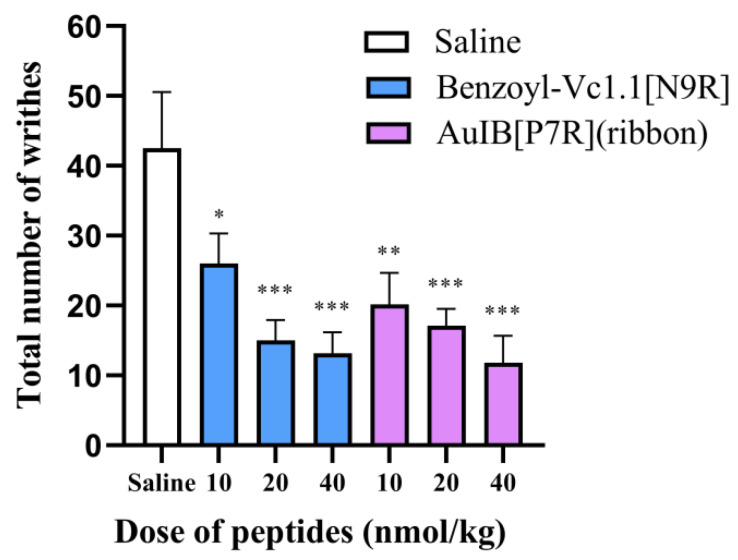
Analgesic activities of AuIB[P7R](ribbon) in the acetic acid writhing model. A total of 30 min after the single administration of Benzoyl-Vc1.1[N9R] (10, 20, 40 nmol/kg, i.p.), AuIB[P7R](ribbon) (10, 20, 40 nmol/kg, i.p.), or saline (i.p.), the writhing number of mice in 15 min was counted 5 min after the administration of acetic acid (1%, 0.4 mL). Data were expressed as mean ± SEM (*n* = 10). * *p* < 0.05, ** *p* < 0.01, and *** *p* < 0.001 vs. the saline group. All data are presented as mean ± SEM.

The analgesic activity was first determined using the acetic acid writhing model. The Vc1.1 variant Benzoyl-Vc1.1[N9R] was selected as the positive control group because it possesses high analgesic activity and acts on GABA_B_R-coupled Ca_V_2.2 [12]. The results show that AuIB[P7R](ribbon) significantly reduces the writhing number (Figure 5) compared to the saline group. Its analgesic activity is similar to Benzoyl-Vc1.1[N9R] at all doses, the writhing numbers are 26.00 ± 4.33, 15.00 ± 2.92, and 13.20 ± 2.98 times for the Benzoyl-Vc1.1[N9R] groups (10, 20, 40 nmol/kg), and 20.20 ± 4.45, 17.11 ± 2.41, and 11.80 ± 3.89 times for AuIB[P7R](ribbon) (10, 20 and 40 nmol/kg), respectively.

### 2.6. AuIB[P7R](ribbon) Also Displays Potent Analgesic Activity in the Rat Partial Sciatic Nerve Injury Model

**Figure 6:**
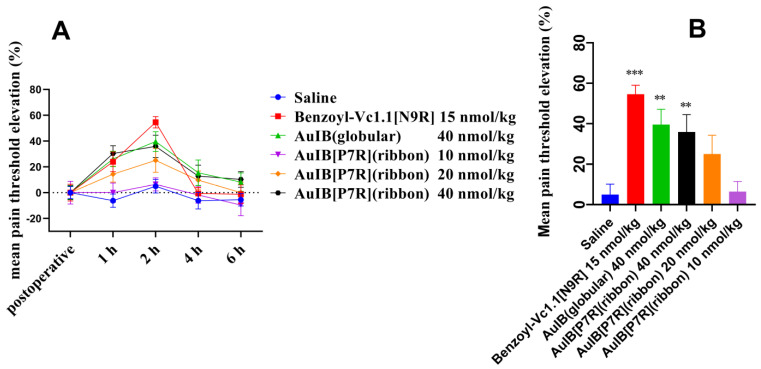
Analgesic activity of AuIB(globular) and AuIB[P7R](ribbon) in the rat PNL model. PNL rats (*n* = 8) were intramuscularly administrated with saline, AuIB(globular) (40 nmol/kg), AuIB[P7R](ribbon) (10, 20, 40 nmol/kg), or Benoyl-Vc1.1[N9R] (15 nmol/kg). (**A**) The line graph shows the mean pain threshold elevation (%) at pre-injection and 1, 2, 4, and 6 h after treatment. (**B**) The bar graph shows the mean pain threshold elevation percentage 2 h after i.m. injection. Data are expressed as mean ± SEM. ** *p* < 0.01; *** *p* < 0.001 compared with saline group.

The analgesic activity of AuIB(globular) and AuIB[P7R](ribbon) were also evaluated by the partial sciatic nerve injury (PNL) model. The results show that AuIB(globular) and AuIB[P7R](ribbon) have potent analgesic activity compared to the saline group. The PTEPs (mean pain threshold elevation percentages) values of each peptide group started to rise and reached the peak 2 h after ipsilateral muscular injection, then decreased to baseline levels around 6 h. Compared to Benzoyl-Vc1.1[N9R] (Figure 6A), AuIB(globular) and AuIB[P7R](ribbon) show a slower decreasing trend. Repeated ANOVA analyses shows no significant difference among Benzoyl-Vc1.1 (15 nmol/kg), AuIB(globular) (40 nmol/kg), and a high dose (40 nmol/kg) of AuIB[P7R](ribbon). A total of 2 h after ipsilateral injection, AuIB[P7R](ribbon) (10, 20, and 40 nmol/kg) increased the PTEPs in a dose-dependent manner, by 6.41 ± 5.11%, 25 ± 9.33%, and 35.90 ± 8.61%, respectively. The PTEPs of Benzoyl-Vc1.1[N9R] (15 nmol/kg) and AuIB(globular) (40 nmol/kg) were 54.60 ± 4.45% and 39.60 ± 7.59% (*n* = 8), respectively.

## 3. Discussion

According to the structure–activity of 4/7-type α-CTxs, a series of AuIB variants at position 4, 5, 9, 10, 11, 12, and 15 were synthesized and evaluated. The results show that the substitutions or additions of one or two residues at the above position with basic or hydrophobic amino acid do not significantly enhance AuIB’s potency to GABA_B_R-coupled Ca_V_2.2. However, the substitution of Pro7 with Arg in AuIB[P7R](ribbon) with the disulfide bridges “1–4, 2–3” results in a 2–3-fold increase in the potency (IC_50_ is 0.74 nM). To our knowledge, AuIB[P7R](ribbon) is the most potent 4/6 type α-CTx targeting GABA_B_R-coupled Ca_V_2.2, and its potency is slightly lower than that of Benzoyl-Vc1.1[N9R], a known inhibitor of GABA_B_R-coupled Ca_V_2.2 [12]. The activity change trend may be related to the rigid structure of AuIB that has several Pro residues in loop1 and shorter amino acid residues in loop2. Interestingly, the further addition of a benzoyl group at N-terminus of AuIB[P7R] did not result in the increase in potency (Table 1), which is different from the results for Vc1.1[N9R], where the addition of benzoyl group at its N-terminus significantly increased the inhibitory activity [12]. The benzoyl group may impair AuIB[P7R] binding to GABA_B_R.

Interestingly, AuIB[P7R](ribbon) displays the greatest potency to GABA_B_R-coupled Ca_V_2.2, but not the globular isomer of AuIB[P7R]. This character is also different from the isoforms of AuIB, in which the globular isomer is more potent than that of the ribbon isomer. In addition, AuIB[P7R](ribbon) displays no apparent inhibitory activity against the nAChRs subtypes at 10 μM. On the contrary, AuIB(globular) exhibits enhanced activity against α3β4 nAChRs compared to the ribbon isomer [29,30], but in the parasympathetic neurons, AuIB(ribbon) shows higher potency against whole nAchRs [31]. Furthermore, our experiments demonstrate that AuIB(globular) exhibits potent inhibitory activity against the GABA_B_R-coupled Ca_V_2.2 (Table 2), but not against its ribbon isomer. To date, only one 4/7-type α-CTx Lt1.3 with disulfide bridges (1–4, 2–3) has been found to target the GABA_B_R-coupled Ca_V_2.2 [21], and AuIB[P7R](ribbon) is the first 4/6 type α-CTx with disulfide bridges (1–4, 2–3) inhibiting this target.

Finally, AuIB[P7R](ribbon) exhibits potent analgesic activity in the acetic acid writhing model and the rat partial sciatic nerve injury model (Figure 5 and Figure 6). Compared to AuIB(globular), AuIB[P7R](ribbon) did not show higher analgesic activity in the acetic acid writhing model at the same dose (40 nmol/kg). This observation could potentially be explained by the compensatory action of AuIB(globular) on α3β4 nAChRs, which is a target for analgesic activity [10]. In addition, 40 nmol/kg AuIB(globular) and AuIB[P7R](ribbon) did not show apparent analgesic activity in the mice hot plate pain model (data not shown).

In summary, we found that a new 4/6 type α-conotoxin AuIB variant AuIB[P7R](ribbon) potently inhibits the GABA_B_R-coupled Ca_V_2.2 with an IC_50_ less than 1 nM, and the most potent isomer is the ribbon, but not the globular isomer. AuIB[P7R](ribbon) exhibits potent analgesic activity in the acetic acid writhing model and the rat partial sciatic nerve injury model. These finding demonstrate that 4/6 type α-CTxs, with the disulfide bridge connectivity “1–4, 2–3,” are also potent inhibitors for GABA_B_R-coupled Ca_V_2.2 and possess potent analgesic activity.

## 4. Materials and Methods

### 4.1. Chemicals and Biological Reagents

The *N*-Fmoc-amino acids, HOBt and HBTU, were purchased from GL Biochem Ltd. (Shanghai, China). Rink resin was obtained from Tianjin Nankai Hecheng S&T Company. Dimethylformamide (DMF), piperidine, dichloromethane, acetonitrile, anhydrous ether, and anhydrous methanol (MeOH) were purchased from Sinopharm Chemical Reagent Co., Ltd. (Beijing, China). Diisopropylethylamine (DIEA), trifluoroacetate (TFA), dithiothreitol (DTT), and triisopropylsilane (TIPS) were purchased from Beijing InnoChem Science & Technology Co., Ltd. (Beijing, China). All chemical reagents were of analytical grade. Fetal bovine serum and DMEM medium were obtained from Gibco (Carlsbad, San Diego, CA, USA).

### 4.2. Animals

Kunming mice (18–20 g, 3–4-week-old, quality license No. 110324220102142031) and Sprague–Dawley rats (200–220 g, 6–7-week-old, quality license No. 11032420102254558) were obtained from SPF (Beijing) Biotechnology Co., Ltd. (Beijing, China), and housed at a relative humidity of 50% under a 12 h light/dark cycle. Food pellets and water were available ad libitum. All experiments were conducted in accordance with the guidelines of the Animal Research Advisory Committee of the Beijing Institutes for Biological Science and conformed to the European Community directives for the care and use of laboratory animals.

### 4.3. Peptide Synthesis

The protected AuIB and its variants were synthesized on Rink resin using the coupling agents HOBt/HBTU and DIEA, as described previously [12]. The peptide-resin (0.05 mmol) is cleaved in the cleavage solution (4.4 mL TFA, 0.25 mL H_2_O, 0.25 g DTT, 0.1 mL TIPS) at room temperature for 3 h to remove the resin and side chain protecting groups. The released linear peptide was filtered, precipitated with cold anhydrous ether at 4 °C for 30 min, and folded in 0.1 M NH_4_HCO_3_ buffer (pH 8.0–8.2) at a concentration of 0.2 mg/mL for 24–48 h at room temperature. After the completion of folding, the folded solution was acidified with acetic acid to a pH of 4.0–5.0. The mixture was then filtered and directly loaded onto a 25 × 250 mm preparative C18 column using a preparative HPLC pump (Waters Delta Prep 4000). The column was washed with buffer A (0.1% TFA in water) at a flow rate of 2.5 mL/min and then with buffer B (0.1% TFA in acetonitrile) at a flow rate of 5 mL/min. The resulting peptide was further purified by semi-preparative RP-HPLC using a Kromasil 10.0 × 250 mm C18 column, and the target peaks were collected and analyzed for purity by HPLC. Confirmation of the correct molecular mass was ascertained by mass spectrometry on a ProFLEXTM-III MALDI-TOF spectrometer. The primary sequences of AuIB and its variants are listed in Table 1.

### 4.4. Disulfide Bond Connectivity Analysis

According to the potency, the disulfide bond connectivity of several AuIB variants folded by one-step oxidation was determined by comparison with peptide folding products of known disulfide connectivity, as described previously [32,33]. Briefly, linear AuIB variants containing an acetamidomethyl (Acm)-protecting group at the C1–C3 or C1–C4 positions were synthesized and then folded by incubation in 0.1 M NH_4_HCO_3_ (pH 8.0) at room temperature for 24–72 h. The folded products were further oxidized with an iodine mixture (30% CH_3_CN, 2% TFA, 68% H_2_O) for 10 min to form disulfide bond connectivity “C1–C3, C2–C4” or “C1–C4, C2–C3,” and then co-analyzed with a one-step folding product using HPLC.

### 4.5. Circular Dichroism (CD) Spectra

CD spectra were measured using a BioLogic Mos 450 spectropolarimeter (Grenoble, France), as described previously [32]. Briefly, 35 μM of peptide in 10 mM of phosphate buffer (pH 7.2) was added into a 0.1 cm path length quartz cell, and three individual scans (190–260 nm) were performed at 1.0 mm intervals with a bandwidth of 1.0 nm.

### 4.6. HEK293T Cell Electrophysiology

The inhibitory activities of AuIB and its variants against GABA_B_R-coupled Ca_v_2.*2* in vitro were determined, as previously described [12,21]. Briefly, HEK293 cells were co-transfected with human GABA_B_R plasmids (0.5 μg GABA_B1_ and 0.5 μg GABA_B2_), rat Ca_v_2.2 plasmids (0.5 μg α1B, 0.5 μg α2δ and 0.5 μg β3), and 0.2 μg of the reporter gene EGFP for 10 min. After incubation at 37 °C in 5% CO_2_ for 48 h, the trypsinized cells were added dropwise to a poly-L-lysine-coated glass coverslip for 30 min, and then 2 mL of culture medium was added and left to incubate for 3 h. Whole-cell voltage clamp recordings were completed using borosilicate glass pipettes at a resistance of ~3 MΩ after being filled with intracellular solution. The intracellular solution was composed of 120 mM K-gluconate, 5 mM MgCl_2_·6H_2_O, 2 mM CsCl, 2 mM MgATP, 0.6 mM Na_2_GTP, 10 mM HEPES, and 5 mM EGTA (pH adjusted to 7.2 with CsOH). The extracellular solution consisted of 90 mM NaCl, 5 mM CsCl, 30 mM TEA-Cl, 10 mM D-glucose, 10 mM BaCl_2_·H_2_O, 1 mM MgCl_2_·6H_2_O, and 10 mM HEPES (pH 7.4). AuIB and its variants were diluted to the appropriate final concentration and applied via perfusion; 10 μM baclofen was used as a positive control. All currents were elicited by a voltage step from a holding potential of −90 mV to a test pulse of +10 mV for 200 ms, with a 10 s interval per sweep at room temperature (20~25 °C) with the Axoclamp 700B patch-clamp. When the peak current of baclofen was inhibited by more than 50%, the drug tested was considered effective, and each current data was repeated for more than 4 cells. The concentration-response data were fitted to the equation: *Y* = *Y*_min_ + (*Y*_min_ − *Y*_max_)/(1 + 10 ^(Log IC^_50_^− X)^ × *h*)), where *Y* (response rate) is I/I_0_, *h* is the Hill coefficient (slope), and IC_50_ is the half-maximal inhibitory concentration. The non-linear regression analysis was performed using GraphPad Prism 8.0 (GraphPad Software, San Diego, CA, USA).

The inhibition of AuIB and its variants against Ca_v_2.2 expressed in HEK293 was also determined using whole-cell voltage clamps [22,34]. The experimental conditions are similar to the tests of GABA_B_R-coupled Ca_V_2.2, except for the lack of GABA_B_R plasmids. In addition, different external bath solutions (20 mM BaCl_2._H_2_O, 135 mM NMDG, 2 mM MgCl_2_.6H_2_O, 10 mM HEPES, pH 7.2) and intracellular solutions (140 mM CsCl, 10 mM NaCl, 10 mM HEPES, and 1 mM EGTA, pH 7.3) were used.

### 4.7. Two-Electrode Voltage-Clamp Recording on Oocytes Expressing nAChRs

Since AuIB[P7R](ribbon) has a significant inhibitory effect on GABA_B_R-coupled Ca_V_2.2, its inhibitory activities on the nAChRs subtypes (α2β4, α3β2, α3β4, α4β2, α4β4, α7, and α9α10) were determined by the two-electrode voltage-clamp technique, as described previously [12,21]; the molar ratio of the cRNA of the subunits per injection was 1:1.

### 4.8. Acetic Acid Writhing Test

Male Kunming mice were randomly divided into seven groups, ten animals per group. Each group received saline, Benzoyl-Vc1.1[N9R] (10, 20, 40 nmol/kg), and AuIB[[P7R](ribbon) (10, 20 and 40 nmol/kg). All peptides and saline were intraperitoneal injected (i.p.) into the animals; the injection volume was 100 μL. A total of 30 min after the peptides or saline injection, acetic acid (1% in saline, 400 μL) was intraperitoneal administrated to each group. After 5 min, the number of abdominal contortions (writhes) was recorded for 15 min. Data are expressed as the total number of writhes. One-way ANOVA was performed, followed by Dunnett’s multiple comparisons test using GraphGad prism 8 (GraphPad Software, La Jolla, CA, USA). All data are presented as mean ± SEM. A p-value less than 0.05 was considered significant.

### 4.9. Partial Sciatic Nerve Injury (PNL) Model Tests

The partial sciatic nerve injury (PNL) model was used to investigate the analgesic activity of AuIB[P7R], as described previously [12,34,35]. The mechanical paw withdrawal threshold (PWT) was tested seven days after surgery by a paw-pressure analgesy meter (Ugo Basile Biological Research Apparatus Co., Comerio-Varese, Italy). Animals with 30%~50% decreases in PWT were selected. The successful PNL model animals were randomly divided into six groups, eight animals per group. Each group received saline, Benzoyl-Vc1.1[N9R] (15 nmol/kg), AuIB(globular) (40 nmol/kg), and AuIB[P7R](ribbon) (10, 20, 40 nmol/kg), respectively. Paw withdrawal thresholds were measured at 1, 2, 4, and 6 h following intramuscular (i.m.) administration close to the injury site at a mid-thigh region in a volume of 100 μL. Data are expressed as mean pain threshold elevation percentages (PTEPs) ± SD. (%PTEP = (Post-drug PWT − Pre-drug PWT)/Pre-drug PWT × 100).

### 4.10. Statistical Analysis

The results of analgesic tests were analyzed using repeated measures of ANOVA, followed by the unpaired t-test through GraphGad Prism 8. All data were presented as means ± SEM. *p* < 0.05 was considered as statistically significant.

## Data Availability

Data is contained within the article and could be provided if requested.
